# Probing Photoluminescence in Perovskite-Based Polymer Nanocomposite Films

**DOI:** 10.3390/polym17172317

**Published:** 2025-08-27

**Authors:** Jack Francis Renaud, Ashlyn Schlabach, Meenakshi Narayan, Evan Davies, Morgan Gillis, Jack Gugino, Nisreen Nusair, Mark P. S. Krekeler, Mithun Bhowmick

**Affiliations:** 1 Department of Mathematical and Physical Sciences, Miami University, Middletown, OH 45042, USA; renaudjf@miamioh.edu (J.F.R.); schlabad@miamioh.edu (A.S.); daviese3@miamioh.edu (E.D.); nusairn1@miamioh.edu (N.N.); 2 Department of Engineering Technology, Miami University, Middletown, OH 45042, USA; narayam3@miamioh.edu; 3 Department of Geology and Environmental Earth Science, Miami University, Oxford, OH 45056, USA; gillism@miamioh.edu (M.G.); guginojp@miamioh.edu (J.G.); krekelmp@miamioh.edu (M.P.S.K.); 4 Department of Mathematical and Physical Sciences, Miami University, Hamilton, OH 45011, USA

**Keywords:** perovskite, quantum dots, polymer nanocomposites, photoluminescence

## Abstract

Polymer nanocomposites incorporating perovskite (PV) nanoparticles have recently emerged as highly promising materials for optoelectronic and photonic devices. In this work, steady-state and time-resolved photoluminescence (PL) were performed in PV-based polydimethylsiloxane (PDMS) nanocomposite films. The steady-state PL measurements revealed linearly increasing emission as excitation intensities ramped up, followed by a saturation. The optical limiting was scalable through the PV concentrations and is likely due to creation of maximum number of electron–hole (e–h) pairs in the system. The presence of a PDMS altered the multi-exponential PL decay significantly, both in terms of underlying mechanism and the associated timescales. The introduction of PDMS changed a 3-component exponential decay of PV into a 2-component mechanism and reduced the total timescale of decay from 16 ns to ~6 ns.

## 1. Introduction

Polymer nanocomposites represent a rapidly advancing area of material science that involves the incorporation of nanoscale fillers into polymer matrices to enhance and tailor the properties of conventional polymers. These composites typically contain fillers such as carbon nanotubes (CNTs), graphene, layered silicates (e.g., montmorillonite), or metal oxide nanoparticles, which can significantly improve mechanical strength, thermal stability, barrier properties, flame retardancy, and electrical conductivity, even at low filler loadings [1,2]. The remarkable enhancements observed in polymer nanocomposites are attributed primarily to the high surface area and strong interfacial interactions of nanofillers with the polymer matrix.

The interest in polymer nanocomposites spans a wide range of industries, including aerospace, automotive, electronics, biomedical devices, and packaging, where performance, weight reduction, and material functionality are critical [3]. Compared to traditional composites with micron-scale fillers, nanocomposites offer a more uniform dispersion and improved interfacial adhesion, which leads to superior property enhancements. However, challenges such as achieving homogeneous filler dispersion, optimizing filler–matrix interactions, and developing scalable, cost-effective fabrication techniques still limit their full commercial potential [4].

Among the different polymer nanocomposite materials, polydimethylsiloxane (PDMS) is a silicon-based organic polymer that has become a cornerstone material in both industrial and research applications due to its distinctive physicochemical properties. Structurally, PDMS consists of repeating –[Si(CH_3_)_2_–O]– units, which confer the polymer with high flexibility, low glass transition temperature, and excellent thermal and oxidative stability. The methyl groups on the silicon atoms create a hydrophobic surface, making PDMS resistant to water absorption and many chemical reagents. These attributes contribute to its use in a wide range of settings, from lubricants and sealants to medical implants and wearable sensors [5]. Due to its optical transparency, biocompatibility, and ease of fabrication via soft lithography, PDMS is widely employed for producing microfluidic devices. It allows for rapid prototyping of lab-on-a-chip systems, which are critical in biological and chemical analysis, including point-of-care diagnostics. Its permeability to gases like oxygen and carbon dioxide further enhances its utility in cell culture applications [6]. Incorporating luminescent nanomaterials such as quantum dots (QDs), rare-earth-doped nanoparticles, carbon dots, and transition metal dichalcogenides (TMDs) into polymer matrices allows for the development of hybrid systems with tunable emission properties, enhanced stability, and processability [7].

Despite many investigations on PDMS-based nanocomposite systems, there is more room for further developments where novel materials could be synthesized with simplified recipes and morphologies. Often these unique size-dependent optical properties of QDs—such as CdSe, ZnS, or PbS—are effectively preserved and enhanced by embedding them in a polymer host. This encapsulation not only improves dispersion and prevents aggregation but also protects the QDs from environmental degradation, thereby enhancing their photoluminescent efficiency and stability [8]. Similarly, the integration of carbon-based nanostructures like carbon dots and graphene quantum dots into polymer matrices has been shown to yield composites with strong, tunable PL and excellent biocompatibility, making them suitable for biomedical applications [9].

Moreover, the photoluminescent behavior of polymer nanocomposites is highly dependent on the interaction between the polymer and the nanofiller. Surface functionalization of nanofillers, the polarity of the polymer matrix, and the dispersion state significantly influence emission intensity, wavelength, and quantum yield [10]. Metal halide perovskites, particularly those with the formula ABX_3_ (where A is an organic/inorganic cation, B is a metal cation like Pb^2+^ or Sn^2+^, and X is a halide), exhibit high photoluminescence quantum yields, tunable bandgaps, and long charge carrier diffusion lengths [11]. However, their inherent instability under moisture, heat, and light exposure has limited their practical use. Embedding perovskite nanoparticles into polymer matrices addresses these challenges by providing mechanical support and environmental protection. The polymer acts as a barrier to moisture and oxygen, enhancing the long-term stability of the perovskite phase without significantly compromising its optical performance. Additionally, such composites can be processed into flexible films, enabling applications in wearable electronics, light-emitting diodes (LEDs), solar cells, and sensors [12]. Common polymers used include poly(methyl methacrylate) (PMMA), polystyrene (PS), and polyvinylidene fluoride (PVDF), or polyvinyl alcohol (PVA) which offer compatibility with solution processing and good transparency.

The performance of perovskite–polymer nanocomposites is highly dependent on nanoparticle dispersion, interface compatibility, and the perovskite crystal phase. Efforts such as surface modification of nanoparticles and in situ growth techniques within the polymer matrix have shown promise in improving uniformity and stability. As research progresses, these hybrid materials are expected to play a key role in the development of next-generation flexible and stable optoelectronic devices. To the best of our knowledge, PL from PDMS films with embedded PV nanoparticles have not been studied for their potential as tunable optoelectronic applications.

In this work, we are reporting steady-state (PL) and time-resolved photoluminescence (PL lifetime) properties of PDMS-based perovskite nanocomposites to investigate the following: (1) the impact of polymer (PDMS) and (2) the effects of polymer to nanoparticle ratio on the optical limiting behavior of the same. The goal is to eventually find a pathway towards more efficient, tunable, economic, and robust materials for optoelectronic device applications.

## 2. Materials and Methods

Oleic acid and oleylamine-coated PV (FAPbBr3) nanoparticle solutions in toluene were obtained from Sigma Aldrich (St. Louis, MO, USA) and were used as is to prepare the nanocomposite samples investigated in this study. The QD solutions came with manufacturer’s specifications of a quantum yield of ~70%, emitting between 525 and 535 nm wavelength, with full width at half maximum (FWHM) ~30 nm. Absorption spectra and PL were previously reported from the PQD film on glass substrate to clarify the excitation/emission characteristics of the film, revealing the 1st exciton at ~525 nm, the emission wavelength at ~ 537 nm, with a FWHM confirmed to be ~23 nm [13]. Dynamic light scattering (DLS) was performed using a Zetasizer nano series (Malvern Instruments, Westborough, MA, USA) to determine the size distribution of nanoparticles in the PV solution.

The polymer nanocomposite samples were prepared in a simple recipe as follows. A two-part silicone elastomer PDMS (Sylgard^TM^ 184) kit, comprising a base and a curing agent, were acquired (Dow Corning Corporation, Midland, MI, USA). An optically clear PDMS solution in liquid phase was prepared by mixing the base and the curing agent with the ratio of 10:1. The PDMS solution was mixed with PV nanoparticle solution (Milipore Sigma, St. Louis, MO, USA) and subsequently vortex mixed for 1 min, resulting in a more viscous solution. The ratio of PDMS vs. PV nanoparticle solution was varied from 0–1.5 mL, as detailed in Table 1. Each solution was then drop-cast onto a microscopic slide to prepare drop-cast films in a sequence of S0 through S6, as listed in Table 1, with S0 denoting a purely PDMS film and S6 representing the nanocomposite film with the highest PV concentration. The drop-cast films were room-temperature cured into solid films for measurements. Once the films were cured, they were inspected using a regular microscope (AmScope LLC., Irvine, CA, USA) to detect any macroscopic aggregation. Using surface profilometry (Dektak 3ST from Bruker Corporation, Billerica, MA, USA), the thicknesses of the films were found to be ~500 μm with 7% or less variance. Although the PL peak position and intensity did not show much dependence on the thickness of the layer or on the surface roughness, no measurements are included in this work from the samples where unusual surface imperfections were visible under the normal microscope.

**Figure 1:**
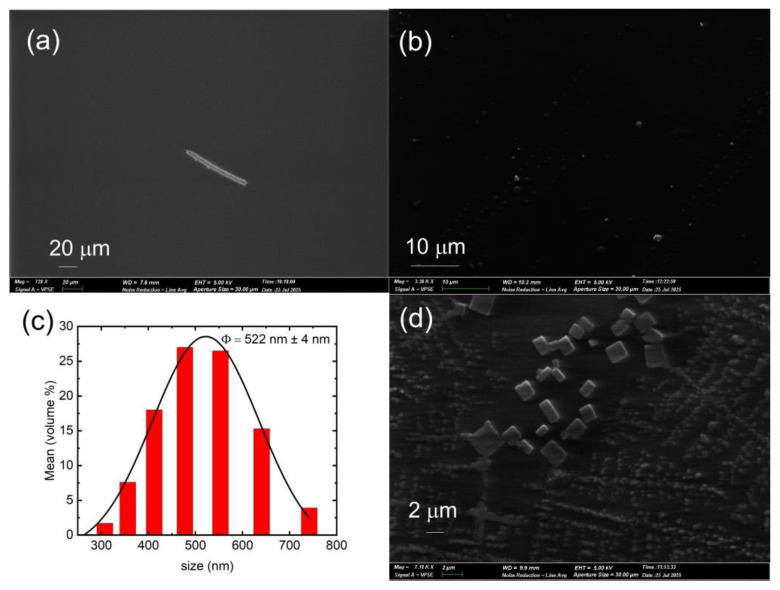
SEM of PDMS layer focusing a deliberate scratch made with a pipette tip for visualization and reference (**a**). The sample S4 in SEM images (**b**), and (**d**), showing PV nanoparticles at different magnifications. The nanoparticle size distribution from DLS measurement is presented with a Gaussian fit for extracting a symmetric error (**c**).

A scanning electron microscope (Bruker Quantax 100 from Bruker Corporation, Billerica, MA, USA) was used to characterize the nanocomposite films, presented in Figure 1.

Photoluminescence measurements were made using a continuous wave, 405 nm laser excitation source from Laserglow Technologies (North York, PA, USA) and a custom-built setup featuring a fiber-coupled spectrometer (Silver Nova from StellerNet Inc., Tampa, FL, USA), a 405 nm notch filter for separating the excitation from the signal, and a collection assembly along the backscattering geometry aided by a focusing lens. The excitation laser intensities could be tuned through a calibrated average power vs. diode current curve, spanning from 0 to 315 mW with 3% or less fluctuations, monitored by a power meter from Laserglow Technologies (North York, ON, Canada). The laser powers were subsequently converted to intensities (W/cm^2^) using the output power (W) and the area of laser beam (cm^2^). The exposure time was set at 5 s, and the average beam area was estimated to be 0.126 cm^2^ for all reported measurements. The standard deviation in beam area, out of 5 measurements, was estimated to be 0.002 cm^2^. Considering the small fluctuation in the beam area, the uncertainty in laser power densities can be estimated as 3% or less.

PL lifetimes were collected using an SP8 confocal multiphoton microscope (Leica Microsystems, Deerfield, IL, USA) using 470 nm excitation with a 20 MHz repetition rate. Finally, all data analysis and fitting were performed using OriginPro 2023 (OriginLab Corporation, Northampton, MA, USA).

## 3. Results and Discussion

The PV nanoparticle solution used in this work was thoroughly characterized previously, and so only a brief summary of characterizations in terms of roughness/aggregation and optical properties will be included here [13]. There was no visible roughness detected in normal microscopes as well as in SEM images.

The polymer film without nanoparticles (S0) showed reasonable smoothness, as can be seen in the SEM image presented in Figure 1a. The feature in Figure 1a is an intentional scratch made in the PDMS film for visual aid. It was difficult to detect aggregation, as indicated in example SEM images presented in Figure 1b,d. The DLS measurement showed broad size distribution, with an average diameter to be ~522 nm ± 4 nm from a fitted Gaussian curve, presented in Figure 1c. Figure 1d shows an example of PV nanoparticles embedded in the polymer matrix, imaged from sample S6, the nanocomposite with highest PV concentration. The size distribution and the higher concentration of nanoparticles are evident and is consistent with the DLS information.

**Figure 2:**
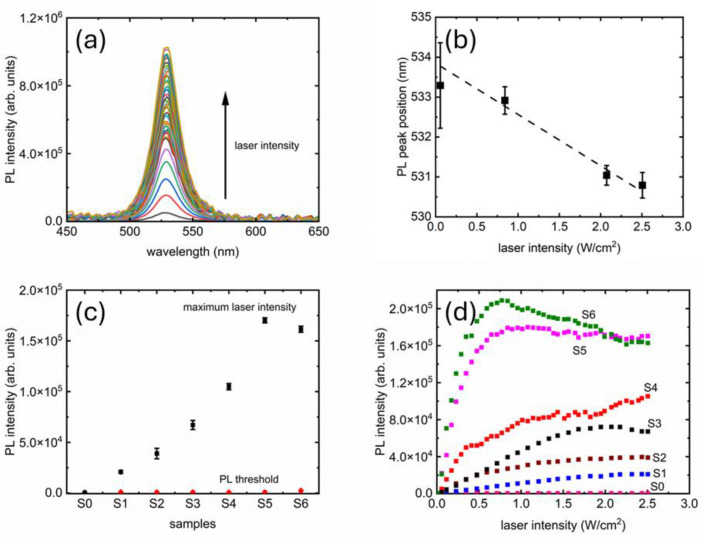
PL from PDMS-based PV nanocomposites: evolution of PL with increasing excitation laser intensity (**a**); consistency of PL peak position as laser intensity increases (**b**); consistency of PL peak intensity for all the samples studied (**c**); and finally, evolution of PL peak intensities for all samples as laser intensity was systematically ramped from PL threshold to maximum (**d**).

The polymer films showed emission at 531 nm ± 0.03 nm with an FWHM of 21.2 nm ± 0.07 nm, corresponding to the excitation threshold of 0.057 W/cm^2^ intensity. Figure 2a shows a representative set of measurements, collected from S4. The PL traces at the highest laser intensities are clearly very close to each other, showing evidence of saturation. The consistency of PL peak positions vs. laser intensity can be seen in Figure 2b. The trend seems to be a weak blueshift. To probe the effect of prolonged exposure to the laser beam, PL measurements were taken for all samples every 10 s, spanning a total duration of 120 s, at the minimum and maximum laser intensities. The results can be seen in Figure 2c, showing reasonable consistency.

**Figure 3:**
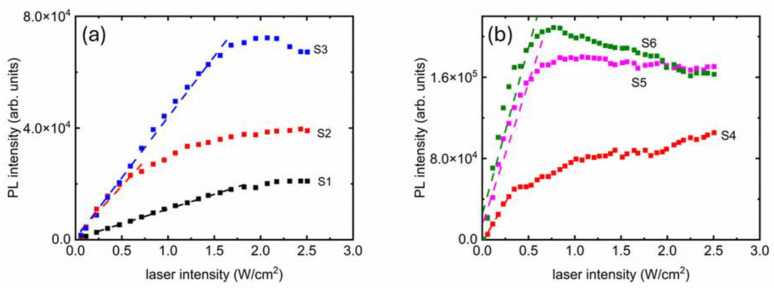
Linear fits (dashed lines) showing consistent increase of PL intensity up to a certain threshold before saturating. The two groups of samples with lower (**a**) and higher (**b**) nanoparticle concentrations displayed similar behavior.

Figure 2d shows the growth of PL peak intensities for all samples, where two distinct areas are visible: (1) a linear growth at the lower intensities up to a certain threshold and (2) a saturation beyond that threshold. The two regions are analyzed further in Figure 3, where linear fits are presented (dashed lines). From Figure 3, we see that the slopes are influenced by the concentration of PV nanoparticles in the PDMS matrix. The samples with higher PV concentrations seem also to have relatively noisier curves. Finally, PL quenching is clearly visible in S6.

**Figure 4:**
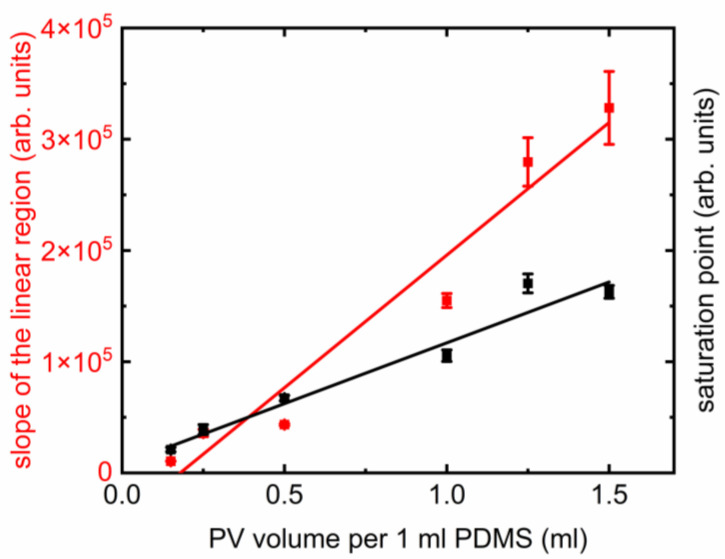
Slope of the linear growth (red symbols) and saturation values (maximum attainable PL intensity) for all samples (black symbols) with their respective linear trends (solid lines).

The maximum PL intensity achievable per unit volume of PV nanoparticles present could be estimated from the saturation values of the curves presented in Figure 2d and Figure 3. The scalability could be better visualized in Figure 4, where the saturation values (indicated as “saturation point” on the right axis) are displayed. The error bars are one standard deviation calculated from the final three data points on the curves from Figure 2d and Figure 3, where the saturation is visible. The trend of saturation points vs. PV nanoparticles per unit volume of PDMS is linear (R^2^ > 0.96), which means we can tune the maximum attainable intensity with the amount of PV nanoparticles. However, it must be noted that the two samples with highest concentrations of PV nanoparticles have overlapping saturation points, and S6 showed quenching. This could be an indication that a very high concentration of PV nanoparticles would not be returning expected PL results.

The rapidity with which PL intensities grew captures the tunability of intensity. The linear fits from Figure 3 shows steadily increasing slopes when the volume of PV nanoparticles per unit PDMS increased. The trend is also linear (R^2^ > 0.94), presented in Figure 4. The error bars of the slopes come from the respective linear fits presented in Figure 3. There is also a difference in stability in the saturation curves. While the lower concentrations (S1, S2, S3) showed smooth linear and nonlinear parts; the higher concentration samples (S4, S5, S6) have noticeable noise.

Nanoparticles and polymer-based nanocomposites are known to have interesting optical limiting behavior [13,14]. While there are several similarities in their PL emission trends under increasing excitation intensities, there are subtly significant differences depending on the morphology of the materials. For polyvinyl alcohol (PVA)-based materials, there was no saturation for emission wavelengths shorter than 650 nm [15]. Polymethyl methacrylate (PMMA)-based nanocomposites showed linear growth similar to PVA-based materials when the nanoparticle density was low but changed into a system where PL saturation and quenching were present, and with higher nanoparticle packing density, the quenching increased even further [14]. The optical limiting is evidently a property from the nanoparticles when they are present without a polymer matrix [13]. However, the nature of temporary photobleaching could be influenced through the choice of substrate on which the films are deposited or through the polymer matrix to which the nanoparticles interact [13,14,15]. It is also possible that the Förster Resonance Energy Transfer (FRET) between closely spaced fluorophores could be present in these PDMS-based samples. We see the effect in the scalability of PL saturation limits and in the presence of quenching for S6. This could explain practically similar PL signal strengths from S5 and S6. The optical limiting or saturation of the PL at the highest excitation intensities could be explained by the way e–h pairs are excited in the system, as is previously seen in many nanoparticle-based materials [13]. During the photo-excitation process, one e–h pair is excited from each of the nanoparticles. At a certain threshold of excitation intensity, it is possible to reach a situation when all available nanoparticles have already absorbed the photons impinging on them. At that point the nanocomposite system must be optically limited and should no longer be able to absorb another photon.

For complex systems with multiple components, it is more likely that there is more than one competing mechanism in play, and a detailed theoretical modeling would be needed to comment on this aspect which is outside the scope of this work, where the focus is on synthesis and optical characterization of a simple nanocomposite material necessary to comment on potential device applications.

**Figure 5:**
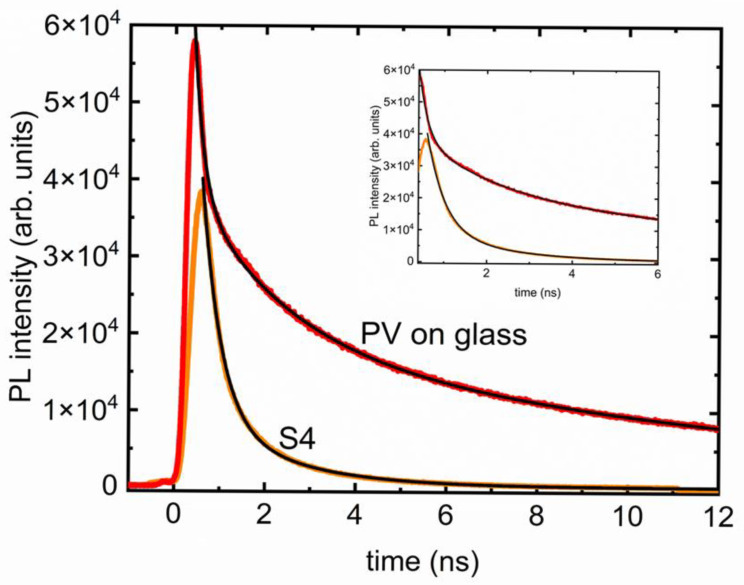
PL lifetime measurements with respective decay curves (black lines) for the PV nanoparticles deposited on glass (red) compared to the same for the sample S4 (orange). The inset provides a clearer picture of the difference in timescales and the presence of multiple components.

A critical parameter for any optoelectronic application is the timescale of PL emission. TRPL measurements in a PV on glass sample, compared with the same in S4, are presented in Figure 5. Photobleaching, either temporary or permanent, could be a result of several factors, including but not limited to the photophysical changes in the absorption or emission spectra, PL lifetime, particle size and density, presence of surface groups, and presence of more particles in the same matrix [16]. To probe the nature of the TRPL curves, both data were modeled using 1-, 2-, and 3-component exponential functions using built-in exponential decay fit routines from OriginPro 2023. The 1-component function did not fit the data well and was discarded, leaving the choice between 2- or 3-component functions. The decision on final applicability was made by several trials of fitting and searching for the optimum number of meaningful time constants. For example, if the 2- and 3-component fits returned comparable $\tau_{1}$ and $\tau_{2}$ and an insignificant $\tau_{3},$ then it would be reasonable to assume that the data followed a biexponential decay. For each finalized choice, it was also checked if the fit accurately captured the timescale of the entire decay. While the PV on glass has 3-component exponential decay, S4 shows 2 components, and an overall much shorter decay time. The PL decay time measurements in the remaining nanocomposite samples were similar to S4.

Notably, the presence of PDMS changed the timescales and decay mechanisms. The drop-cast film of PV on glass sample (solid red line) needed a 3-component exponential decay, shown by the solid black line in Figure 5, of the form

(1) $$I\left(t\right)=I_{0}+{A_{1}e}^{-t/\tau_{1}}+{A_{2}e}^{-t/\tau_{2}}+A_{3}e^{-t/\tau_{3}}$$

where $I\left(t\right)$ is the PL intensity vs. time *t*, $I_{0}$ is the detector background signal, *A*_i_ are fit parameters, and *τ*_i_ are the time constants associated to the three components. The PV on glass decay curve yielded $\tau_{1}=0.2\;ns$, $\tau_{2}=2\;ns$, and $\tau_{3}=14\;ns$, making the whole decay last for more than 16 ns, whereas the polymer nanocomposite sample (S4) curve from Figure 5 could be efficiently fit with a 2-component exponential decay of the form

(2) $$I(t)={{I_{0}+A}_{1}e}^{-t/\tau_{1}}+{A_{2}e}^{-t/\tau_{2}},$$

yielding $\tau_{1}=1.2\;ns$ and $\tau_{2}=4.9\;ns,$ making the whole decay time ~6.1 ns. The uncertainty in these fits is less than 0.2%, which can be used as a limit while comparing the time constants obtained from the fits. Evidently, the first components are the fastest decaying part of the intensity. It is noteworthy that this decay time is significantly longer in S4 compared to the PV-only sample. The second components $\left(\tau_{2}\right)$ are also different, although it could be because of the fundamentally different pathways, since there is no third component present in the PL decay of S4. The total decay times are very different (16 ns in PV vs. 6.1 ns in S4), showing clear evidence of impact due to presence of PDMS matrix. PV nanoparticles by themselves on a substrate previously showed multi-exponential decay times with short and long components. The decay could strongly be altered by the polymers, as reported previously [17,18,19,20,21]. However, the samples studied here are much simpler yet robust, where no stabilizing agent such as hexadecylamine was necessary [22].

It is important to comment on possible application routes in light of the results presented above. Polymer-based nanocomposites are known to degrade from continuous illumination [16]. Any practical light emitting applications would require robustness, a promising aspect that could be explored further in PDMS-based materials discussed in this work. Chemical and biological sensing, photodynamic therapy, and noninvasive biomedical applications seem to align with these properties as well. It has been shown that molecularly imprinted polymers (MIPs) with controllable PL could be used for novel chemosensing applications [23]. It was also revealed that PL decay is a more sensitive method and is preferrable for future bioimaging [23]. The tunability in timescales in nanocomposites studied here is a promising similarity that could be explored further. TRPL studies have also been employed in drug delivery applications where the sensitivity of PL decay was found to be dependent on the environment, helping the release based on distinguishable lifetimes [24]. Bright quantum emitters using long and short timescales of PL decay in PV nanocrystals have been proposed recently [25]. Complex polymeric systems with FRET with photobleaching properties are excellent candidates for light harvesting applications through electroluminescence [26]. The nanocomposites studied here have better FWHM (~21 nm) compared to previously reported materials for similar purposes (~50–100 nm), which would make the nanocomposites applicable for light-emitting applications [26]. Fluorescence lifetimes in the order of ~10 ns, similar to the timescales of TRPL decays in Figure 4 in PMMA-based nanomaterials, were found to be promising in flow cytometry, particularly to increase the degree of multiplexing [27]. These timescales are tunable through water content of the host matrix which can be achieved by incorporating them into a solid host, creating a polycrystalline nanocomposite, creatively made from inorganic or organic nanomaterials [28,29,30,31,32]. Most, if not all, materials explored previously needed a more complex synthesis recipe to achieve clean morphologies and reasonably consistent emission behavior. The PV-PDMS nanocomposites, despite their simplicity, have shown sufficient potential for applications and should be explored further. Possible directions for future studies could be a theoretical modeling of the material system for deeper insight into the PL mechanisms, an investigation of functionalized polymer matrix-induced stabilization pathways or nontoxic nanoparticle integration such as carbon dots for all-organic, all-optical bistable devices or controllable photonic switch applications.

## 4. Conclusions

In this work, steady-state and time-resolved PL from PV-based PDMS nanocomposite films with varying PV concentrations, along with one without any polymer matrix films were investigated. The main results presented in this work are scalable PL intensities from the samples controlled by PV concentration, linear growth of PL with increasing excitation laser intensities, a saturation and/or quenching at the highest laser intensities, essentially proving presence of optical limiting in all samples, and finally, an alteration of PL decay mechanism in presence of the PDMS host, resulting in faster PL decay in nanocomposite films. The scalability of emission intensities strongly depended on PV concentrations. The linear growth as well the saturation, or optical limiting behavior is likely due to the exhaustive nature of e–h creation, typical in QD systems. The change of PL decay timescales could be explained by the sensitivity of PV nanoparticles to the host material (in this case PDMS) and the amount of water present. Although both PV and the PDMS nanocomposites showed multicomponent exponential decays, the nanocomposites showed a 2-component TRPL, in contrast of a 3-component decay present in PV, another possible effect of having a hard matrix such as PDMS in the host.

## Figures and Tables

**Table 1:** List of all samples studied with their respective details.

Samples	PDMS Volume (mL)	PV Volume (mL)
S0	1.00	0.00
S1	1.00	0.15
S2	1.00	0.25
S3	1.00	0.50
S4	1.00	1.00
S5	1.00	1.25
S6	1.00	1.50

## Data Availability

The original contributions presented in this study are included in the article. Further inquiries can be directed to the corresponding author.
